# Unique lentivirus infecting feral simians from forests of Rajasthan, India

**DOI:** 10.1186/1742-4690-8-S1-A212

**Published:** 2011-06-06

**Authors:** Jayashree S Nandi, Anil K Chhangani, Surendra M Mohnot, Felipe Diaz-Griffero

**Affiliations:** 1 Dept. of Microbiology and Immunology, Albert Einstein College of Medicine, NY, 10461, USA; 2 Dept. of Microbiology and Cell Biology, Indian Institute of Science, Bangalore, 560012, India; 3 Dept. of Zoology, JNV University, Jodhpur, Rajasthan, India; 4 Primate Research Centre, Jodhpur, Rajasthan, India

## 

Lentivirus infection of wild rhesus macaques (Macaca mulatta) and langurs (Semnopithecus entellus) from forests of Rajasthan was suspected based on testing of simian plasma samples by commercial HIV-1 WB in 1998. Though simians have a religious connotation in India, aggressive monkey bites are frequently reported from urban and rural areas due to habitat fragmentation of wild simians. Blood samples from 34 feral rhesus macaques and 9 langurs were collected recently from forests of Rajasthan. Plasma samples were tested using monoclonal antibody against capsid proteins of SIVmac (p27) and HIV-1 (p24). Mitochondrial gene analyses confirmed the species of origin. Integrated provirus was detected by Alu-LTR amplification. Lentiviral genes, gag, pol, env, nef, vif, vpr and LTR were amplified and sequenced. Viral load assay was performed independently. Virus purified from simian plasma by ultracentrifugation was used to infect human PBMC, H9 (CD4+, CXCR4+) and U87 (CD4+, CCR5+) cell lines. Virus replication in vitro was detected by p24 antigen capture ELISA and microscopic syncytia formation. Phylogenetic analyses of viral sequences demonstrated intriguing homology to subtype B HIV-1 rather than any known SIV. Significant sequence divergence from HXB2 existed in different regions of viral genome except for conserved pol (RT) region, ruling out inadvertent laboratory contamination. Serological activity was observed against HIV-1 p24 but not SIVmac p27. Reverse transmission of subtype B HIV-1, which is transmitted in north India from infected humans to feral simians due to bleeding monkey bite followed by horizontal spread of the infection in troops of wild simians is proposed.

